# Total Arch Replacement with Ascyrus Medical Dissection Stent Versus Frozen Elephant Trunk in Acute Type A Aortic Dissection: A Meta-Analysis

**DOI:** 10.3390/jcm14145170

**Published:** 2025-07-21

**Authors:** Massimo Baudo, Fabrizio Rosati, Michele D’Alonzo, Antonio Fiore, Claudio Muneretto, Stefano Benussi, Lorenzo Di Bacco

**Affiliations:** 1 Department of Cardiac Surgery Research, Lankenau Institute for Medical Research, Main Line Health, Wynnewood, PA 19096, USA; 2 Department of Cardiac Surgery, Spedali Civili di Brescia, University of Brescia, 25123 Brescia, Italy; rosati.fabri@gmail.com (F.R.); claudio.muneretto@unibs.it (C.M.); stefano.benussi@unibs.it (S.B.); lorenzo.dibacco@hotmail.it (L.D.B.); 3 Department of Cardiac Surgery, Poliambulanza Hospital Brescia, 25124 Brescia, Italy; dalonzomi@gmail.com; 4 Department of Cardiac Surgery, Hôpitaux Universitaires Henri Mondor, Assistance Publique-Hôpitaux de Paris, F-94000 Créteil, France; antonio.fiore@aphp.fr; 5 CEpiA Team, IMRB U955, Inserm, Université Paris Est Créteil, F-94000 Créteil, France

**Keywords:** frozen elephant trunk, ascyrus medical dissection stent, fet, amds, aortic surgery, cardiac surgery

## Abstract

**Background**: Acute Stanford Type A aortic dissection (ATAAD) often requires total arch replacement (TAR) with frozen elephant trunk (FET) to address entry tears and support aortic remodeling. In select cases, AMDS may provide a simpler option. The present meta-analysis aims to compare surgical outcomes between these two approaches. **Methods**: A comprehensive search in the Pubmed, ScienceDirect, SciELO, DOAJ, and Cochrane library databases was performed until February 2025. We included studies that reported the outcomes of patients with ATAAD undergoing TAR with AMDS or FET. To enable a meaningful comparison, we only included FET studies where patients met the same inclusion criteria as those with the AMDS. **Results**: Thirty-eight articles met our inclusion criteria, with a total of 319 patients in the AMDS group and 4129 in the FET group. Patients undergoing an AMDS procedure experienced significantly higher bleeding requiring surgery (21.2% vs. 6.4%, *p* < 0.001) and a higher hospital mortality (14.5% vs. 10.0%, *p* = 0.037) compared to FET. The individual patient data of 1411 patients were constructed. Overall survival at 1 and 3 years was 81.9% ± 3.3% vs. 88.8% ± 0.9% and 81.9% ± 3.3% vs. 85.2% ± 1.0% between AMDS and FET, respectively. A flexible parametric survival model demonstrated a significant mortality drawback for AMDS compared to FET up to 31 days, beyond which the difference was no longer evident. **Conclusions**: The comparison between AMDS and FET for ATAAD treatment remains debated, with FET favored for its lower mortality and stronger long-term evidence. AMDS, as a newer technique, shows promise but lacks sufficient data to confirm its safety and efficacy.

## 1. Introduction

Acute Stanford Type A aortic dissection (ATAAD) still represents a surgical challenge carrying a significant risk of morbidity and mortality [1]. It is of paramount importance to surgically excise the entry tear by replacing the dissected aorta with a vascular graft. The simplest surgical management of ATAAD considers the replacement of the ascending aorta. Nevertheless, in ATAAD DeBakey Type 1, the presence of an entry tear or the extension of the false lumen into the aortic arch and the descending thoracic aorta poses patients at an increased risk of further complications, thus, more extensive approaches, such as total arch replacement (TAR), have been advocated [2,3].

In selected cases, aortic arch repair by the means of frozen elephant trunk (FET) procedures gained prominence due to the capability of inducing true lumen stabilization and favoring false lumen thrombosis during TAR [4]. Moreover, it may facilitate the deployment of further endovascular graft at the level of the descending aorta. Several grafts are currently available for FET procedures, such as the Thoraflex (Vascutek Ltd., Renfrewshire, Scotland, UK) and E-vita (Artivion Inc., Kennesaw, GA, USA) in Western countries, or the J Graft FROZENIX (Japan Lifeline, Tokyo, Japan) and Cronus prosthesis (Microport Medical, Shanghai, China) in Eastern countries [5].

Additionally, a recently described novel tool was introduced into the surgical armamentarium. The Ascyrus Medical Dissection Stent (AMDS; Ascyrus Medical, Boca Raton, FL, USA) is a partially uncovered aortic hybrid graft that consists of a tubular nitinol frame attached to a proximal Teflon fabric cuff for proximal anastomosis. During ATAAD DeBakey Type 1, the AMDS is fixed with its Teflon cuff at the level of the distal (open) anastomosis, while the stent is deployed into the true lumen at the level of the arch and beyond. The aim is to re-expand the true lumen, promote aortic arch remodeling, reduce the risk of distal malperfusion, and reduce the risk of distal anastomotic new-entry tear (DANE) without increasing surgical complexity. It should be remembered though that the presence of an entry tear at the level of the arch may contraindicate the use of this device.

Several studies have highlighted the potential of the AMDS device to restore perfusion in supra-aortic vessels affected by dissection. Some observed the complete resolution of malperfusion in these branches [6,7], while others reported successful reperfusion in 95% of previously compromised vessels [8]. These outcomes suggest that AMDS may offer a targeted solution to supra-aortic involvement, potentially reducing the need for extensive arch reconstruction, as seen in TAR. However, improvements in branch vessel perfusion have not consistently aligned with neurological outcomes [7].

In terms of aortic remodeling, AMDS has shown encouraging results in the arch and supra-aortic regions, yet its impact is less favorable in the distal aorta [9]. Furthermore, current data are insufficient to draw firm conclusions about the medium- and long-term remodeling capacity of AMDS.

The present meta-analysis aims to compare surgical outcomes between these two approaches.

## 2. Materials and Methods

### 2.1. Protocol and Registration

This review is registered with the PROSPERO database of systematic reviews [10] under the ID CRD42023433762. Since this study involves the analysis of previously published data and does not include individual patient involvement, neither research ethics board approval nor patient consent was required. The data that support the findings of this study are available from the corresponding author upon reasonable request.

### 2.2. Search Strategy

**Figure 1:**
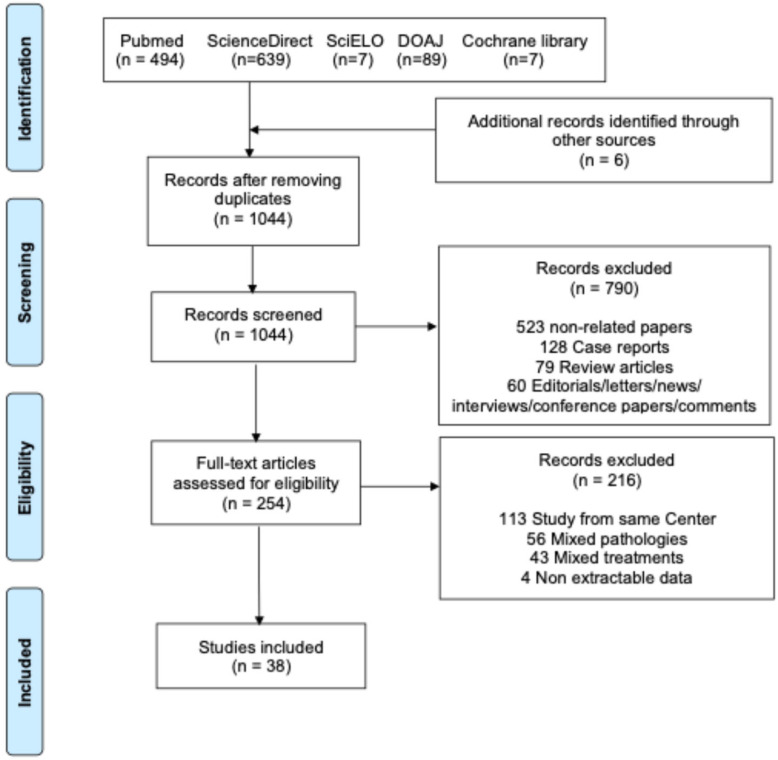
Preferred Reporting Items for Systematic Reviews and Meta-Analyses (PRISMA) of included studies.

This systematic review was conducted according to the Preferred Reporting Items for Systematic Reviews and Meta-Analyses (PRISMA) guidelines [11]. The PRISMA flow diagram is displayed in Figure 1. We conducted a comprehensive search in the Pubmed, ScienceDirect, SciELO, DOAJ, and Cochrane library databases until February 2025. We aimed to identify publications reporting on patients undergoing TAR with AMDS or FET in ATAAD. The search strategy details are outlined in Table S1. Additionally, we scrutinized the reference lists of studies and reviews to uncover additional relevant articles (i.e., backward snowballing). The screening process for inclusion was independently carried out by two authors (M.B. and M.D.). In instances of disagreement, a consensus was achieved with the input of a third senior author (L.D.B.).

### 2.3. Inclusion and Exclusion Criteria

We included studies that reported the outcomes of patients with ATAAD undergoing TAR with AMDS or FET. Studies were excluded in cases where the population included aneurysm or other non-urgent aortic pathologies, surgery other than TAR, or techniques other than AMDS or FET were performed. Supra-aortic vessel stent branching or modified/simplified FET techniques were also excluded. To enable a meaningful comparison between the two populations, we only included FET studies where patients met the same inclusion criteria as those with AMDS. Consequently, FET studies reporting entry tears at the level of the arch were also excluded.

Furthermore, case reports, reviews, abstract, presentations, comments, and non-English papers were also discarded. In cases of multiple publications from the same institution, the study period was assessed, and the largest sample size study was included if overlap existed.

### 2.4. Data Extraction

Data extraction was performed using Microsoft Office 365 Excel software (Microsoft, Redmond, WA, USA). Information regarding the study period, study center, country, and sample size was extracted. Subsequently, patients’ characteristics, postoperative outcomes, and follow-up results were abstracted.

Encountered variances in reported information among cases were anticipated, and to some extent, each article introduced distinct variables not found in other publications. Consequently, interpretation was necessary for addressing missing data related to certain variables. The denominators for specific variables were indicative of their respective percentage values. When presenting this data, the denominators relied on explicit mentions of variable presence or absence, or on reasonable inference when applicable.

When accessible, individual patient data (IPD) was collected from the Kaplan–Meier plots. Following the methodology outlined by Liu et al. [12], a two-part process was employed for data extraction. The initial step involved digitizing the Kaplan–Meier curves using specialized software called WebPlotDigitizer (available at https://automeris.io/wpd/ accessed on 23 April 2025). This software allowed for the definition of axes, and through mouse clicks, specific points on the curve were selected, enabling the extraction of raw data coordinates for time and the overall survival probability for each treatment group within each Kaplan–Meier plot. To ensure accuracy, the digitized Kaplan–Meier curves were visually cross-referenced with the original curves. The collected Kaplan–Meier data from various studies were consolidated in the study database. In the subsequent phase, the raw data coordinates from the initial step were processed. This processing involved incorporating the numbers at risk at specific time points and/or the total patient count, and the R package “IPDfromKM” ver. 0.1.10 was employed to reconstruct IPD. Ultimately, the reconstructed IPD from all included studies were merged to form the comprehensive study dataset.

### 2.5. Critical Appraisal and Outcomes of Interest

The quality of included non-randomized studies was assessed through the ROBINS-I tool [13]. For randomized clinical trials (RCTs), the RoB 2 [14] was utilized.

The primary endpoint was mortality, while the secondary endpoints encompassed postoperative complications, neurological in particular (i.e., cerebrovascular accidents and spinal cord ischemia).

### 2.6. Statistical Analysis

For postoperative outcomes, pooled event rates (PERs) or means (PEMs) were calculated, while late outcomes were assessed using incidence rates (IRs) to accommodate the different follow-up durations among studies. Poisson regression modeling was used in this case, assuming a constant event rate. The total person-time of follow-up was calculated from the total number of events and mean follow-up time. A log transformation to model the overall IR was used.

In all analyses, studies were weighted by the inverse of the variance of the estimate for that study, and between-study variance was estimated with the DerSimonian–Laird method with a random effects model. All results were presented with a 95% Confidence Interval (CI). Studies with zero occurrences were included in the meta-analysis, and zero cell frequencies were adjusted using a treatment arm continuity correction. Equivalence hypothesis testing was set at a two-tailed 0.05 significance level. Heterogeneity was assessed using the Cochran Q test, with I^2^ values.

Meta-regression was performed to investigate possible predicting factor for hospital mortality. The results are expressed as odds ratio (OR), 95% CI and *p*-value.

IPD were analyzed using Kaplan–Meier survival curves, and differences between groups were assessed with the log-rank test. Hazard ratios (HRs) with 95% CIs were estimated using Cox proportional hazards regression. The proportional hazards assumption for each Cox model was evaluated using the Grambsch–Therneau test and diagnostic plots derived from Schoenfeld residuals. If the assumption of proportional hazards was violated, a flexible parametric survival model (also known as the Royston–Parmar or generalized survival model) incorporating B-splines and 95% CIs was employed, as these models do not rely on the proportional hazards assumption and are capable of modeling diverse hazard functions.

All analyses were carried out using R, version 4.4.3 (R Project for Statistical Computing, Vienna, Austria), and RStudio version 2025.05.0+496. The “meta” package was employed for the meta-analysis.

## 3. Results

### 3.1. Study Selection and Characteristics

The systematic review process is outlined in Figure 1. The literature search identified 1316 potentially eligible studies. Six additional articles were identified through backward snowballing. After the removal of duplicates, 1044 studies were screened. Among these, 254 full=text articles were assessed for eligibility. Thirty-eight articles [6,15,16,17,18,19,20,21,22,23,24,25,26,27,28,29,30,31,32,33,34,35,36,37,38,39,40,41,42,43,44,45,46,47,48,49,50,51] met our inclusion criteria with a total of 4448 patients, 319 in the AMDS group and 4129 in the FET group. Publication years ranged from 2010 to 2025, and the sample size ranged from 8 to 544 patients. Details of the individual studies are shown in Table S2. The papers included 4 propensity-matched studies, 2 prospective studies, and 32 observational studies. The critical appraisal of the included studies is displayed in Table S3. The baseline patients’ characteristics are shown in Table 1. Patients undergoing AMDS interventions were significantly older (*p* < 0.001), were more often females (*p* = 0.005), were less likely (contraindication) to have connective tissue diseases (*p* = 0.001), and suffered less from hypertension (*p* = 0.014), but they presented more chronic renal disease (*p* < 0.001), a higher BMI (*p* = 0.023), and acute neurological deficit (<0.001) when compared to FET.

### 3.2. Meta-Analysis of the Outcomes

Intraoperatively, patients in the AMDS group were operated at a significantly higher hypothermic temperature (PEM 27.2 °C [95%CI: 26.6–27.8] vs. 23.9 °C [95%CI: 22.9–25.0], *p* < 0.001), had higher concomitant valve sparing root replacement rates (31.8% [95%CI: 9.7–66.7] vs. 3.2% [95%CI: 1.8–5.7], *p* = 0.001), higher concomitant root repair rates (3.6% [95%CI: 0.3–35.8] vs. 0.9% [95%CI: 0.3–2.5], *p* = 0.049), and a longer circulatory arrest time (54 min [95%CI: 30–98] vs. 29 min [95%CI: 24–35], *p* = 0.047) compared to FET. No other intraoperative significant differences were noted between the two groups (Table 2).

During the postoperative course, patients undergoing an AMDS procedure experienced significantly higher bleeding requiring surgical revision (21.2% [95%CI: 14.5–29.9] vs. 6.4% [95%CI: 4.1–10.1], *p* < 0.001) and a higher hospital mortality (14.5% [95%CI: 11.0–18.9] vs. 10.0% [95%CI: 8.1–12.3], *p* = 0.037) compared to FET. Nevertheless, the AMDS patients reported a shorter length of hospital stay (14.8 days [95%CI: 13.3–16.4] vs. 19.2 days [95%CI: 17.3–21.4], *p* < 0.0001). No other postoperative significant differences were observed (Table 3).

In univariable meta-regression, no associations were noted in both groups (Table 4).

### 3.3. Individual Patient Data Analysis

**Figure 2:**
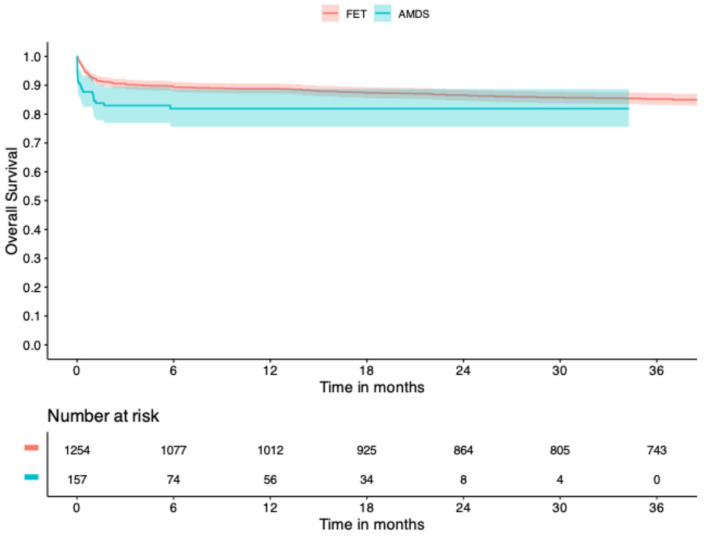
Kaplan–Meier-derived overall survival between AMDS and FET. The two groups showed variable risk of mortality over time, and no single hazard ratio could be calculated. AMDS = Ascyrus Medical Dissection Stent; FET = frozen elephant trunk.

**Figure 3:**
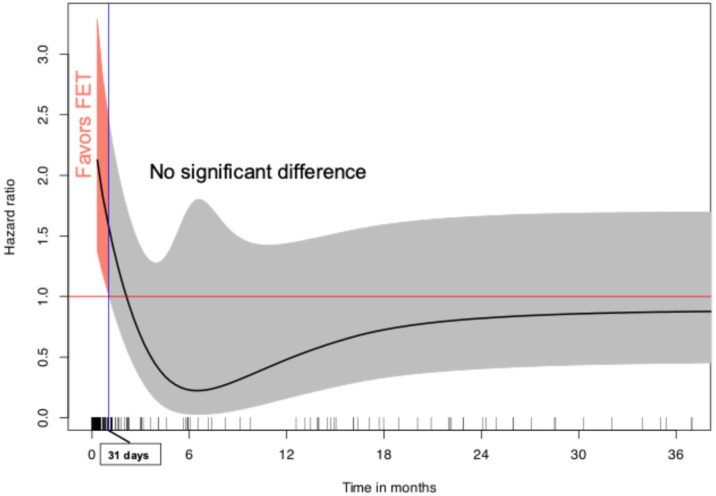
Hazard ratio trend over time for overall survival. FET was associated with an early (31 days) survival advantage compared to AMDS, after which no significant difference was noted between the two groups. FET = frozen elephant trunk.

Fourteen studies among the included papers presented a Kaplan–Meier curve representing overall survival. The IPD of 1411 patients were constructed, 157 patients in the AMDS group and 1254 in the FET group. Overall survival at 1 and 3 years was 81.9% ± 3.3% vs. 88.8% ± 0.9% and 81.9% ± 3.3% vs. 85.2% ± 1.0% between AMDS and FET, respectively, as shown in Figure 2. The Schoenfeld individual test reported a violation of hazard proportionality (*p* < 0.001), Figure S1. Figure 3 illustrates the variation in HR over time from the generalized survival model, demonstrating a significant mortality drawback for AMDS compared to FET up to 31 days, beyond which the difference was no longer evident.

## 4. Discussion

The findings of this meta-analysis suggest notable differences between patients undergoing aortic surgery with AMDS and those treated with TAR using the FET technique. First, the two groups presented with distinct preoperative profiles. Intraoperatively, AMDS procedures were performed at higher hypothermic temperatures, were associated with longer durations of circulatory arrest, and differences were also observed in aortic root management strategies. Postoperatively, AMDS was linked to higher rates of bleeding and early mortality, despite a shorter hospital length of stay. Importantly, overall survival was not proportional over time: IPD analysis revealed increased early mortality within 31 days in the AMDS group, after which survival curves remained parallel, without a statistically significant difference compared to the FET group.

There is an ongoing debate regarding the optimal surgical approach for ATAAD, specifically, whether to opt for a tear-oriented hemi-arch replacement or pursue early TAR. Early TAR is increasingly supported for ATAAD due to its favorable long-term outcomes and comparable short-term mortality [52,53]. Both hemi-arch repair with AMDS and TAR with FET offer effective single-stage hybrid alternatives to classical hemi-arch repair, emphasizing the value of early arch intervention. Among these, FET has demonstrated consistently excellent short- and long-term outcomes and shows broad clinical adoption, supported by strong evidence [54,55]. In contrast, clinical experience with AMDS remains limited, with inconsistent study results and inferior perioperative outcomes compared to FET, making it difficult to draw firm conclusions about its efficacy so far [9].

While FET remains the gold standard for extensive arch repair, especially in cases with malperfusion, it requires specialized surgical expertise and may not be feasible in emergency settings. In such cases, AMDS implantation offers a practical alternative to extend the benefits of hemi-arch repair, provided that there are no contraindications [56]. However, careful preoperative planning is essential, as entries in supra-aortic vessels can still lead to false lumen perfusion and aortic growth. AMDS is indicated for patients with DeBakey type I aortic dissection, particularly when the primary entry tear is located in the ascending aorta or aortic root. It is contraindicated in cases involving aneurysms or entry tears in the aortic arch, descending aorta, or supra-aortic vessels, as well as in patients with connective tissue disorders such as Marfan syndrome or a known allergy to nickel (nitinol). For these reasons, the present analysis made every effort to include only those FET studies whose patient populations and clinical scenarios would also have been eligible for AMDS implantation [56].

It has been previously reported that AMDS demonstrated poorer perioperative outcomes compared to FET [9]. Our findings support this observation, with the AMDS group showing higher rates of reoperation for bleeding and early mortality. While the reduced length of hospital stay in the AMDS group may appear advantageous, it may indirectly be attributable to a higher in-hospital mortality rate, which lowers the overall average duration of hospitalization. This trend was further confirmed through Kaplan–Meier-derived IPD analysis, which revealed a survival advantage for FET within the first 31 days postoperatively, followed by no significant difference in long-term mortality. These results should be interpreted considering that despite the fact that surgical risk scores could not be analyzed, the AMDS group exhibited a significantly higher baseline risk profile, characterized by an older age, increased prevalence of chronic kidney disease, and a higher incidence of acute preoperative neurological deficits. These differences suggest that patients selected for AMDS may represent a more clinically compromised population compared to those undergoing FET. This disparity in baseline characteristics could confound outcome comparisons and may partially explain the differences observed in postoperative morbidity and mortality. Another important point to consider is that the FET group reflects a more mature surgical experience, with the learning curve largely overcome, whereas AMDS is a relatively new device still in the early stages of clinical adoption. No specific predictors were identified in either group.

AMDS was developed to enhance outcomes following hemi-arch repair in ATAAD, particularly by addressing persistent false lumen flow, a major contributor to distal aortic events and reinterventions [57]. Distal anastomotic new entries (DANEs) after standard hemi-arch replacement can reach nearly 40–70% of patients and are strongly linked to aortic growth and adverse outcomes [58]. However, the very limited description of such an outcome (three AMDS studies, and none in the FET group) did not allow us to analyze DANEs in the present study. Indeed, DANEs after ATAAD repair can lead to persistent false lumen pressurization, aortic expansion, and the need for reintervention, affecting long-term outcomes [59]. While TAR may reduce these risks, it carries the risk of high short-term morbidity in low-volume centers. Recent studies on AMDS have shown promising early and mid-term results, including significantly lower rates of DANEs, greater false lumen thrombosis, and improved true lumen expansion in the aortic arch and stented descending aorta [22,60]. However, despite these benefits, no differences in distal aortic growth or late reintervention rates were observed between AMDS and standard hemi-arch repair. Thus, while AMDS appears to promote favorable early aortic remodeling, especially in the arch, long-term data are still needed to confirm its impact on reintervention and aortic remodeling beyond the stented segment.

The present results were mostly retrieved from tertiary high-volume centers expert in aortic surgery. It has been previously demonstrated that hospital outcomes are dependent on the patient’s volume in many different cardiothoracic surgeries, also because these centers generally offer the best technology and trained personnel [61,62,63]. In particular, an analysis of national data from the STS Adult Cardiac Surgery Database revealed a clear association between hospital surgical volume and outcomes in ATAAD [61]. Even after adjusting for surgeon and perioperative factors, lower-volume centers had higher mortality rates. Notably, the majority of U.S. hospitals fell into the lowest-volume quartile, with surgeons performing a median of just one ATAAD case per year over four years. While major morbidity remains high across all volume levels, regardless of hospital experience, higher-volume centers tend to perform a greater proportion of complex procedures, suggesting a link between institutional specialization and case complexity.

For this reason, the use of AMDS should be limited to controlled protocols or highly specialized centers until more robust evidence is available.

### Strengths and Limitations

To the best of our knowledge, this is the first meta-analysis that gathered the different outcomes between AMDS and FET in patients undergoing TAR for ATAAD. Moreover, this study conducted an individual patient data analysis through Kaplan–Meier-derived data. This methodology offers a key advantage over traditional meta-analyses, which assume proportional hazards and, therefore, may obscure meaningful changes in relative risk over time.

On the other hand, the current paper has some limitations. First, only non-randomized trials were included in the analysis, thus adding a potential risk of bias due to confounding and selection of data to the analysis. Indeed, differences in the baseline patients’ characteristics were present between the two groups, probably reflecting the different institutional surgical strategies. The different availability of the two techniques generated a significant difference in the number of patients in the compared groups. Malperfusions were not always reported and, if so, the modality of description differed among studies. Device-related adverse events were assessed; however, complete data were only available for the AMDS group. To avoid biased or unbalanced reporting, these results were omitted from the analysis. Furthermore, the very limited description of DANEs did not allow us to analyze such an outcome in the present study. Considering the clinical importance of this outcome, the lack of the analysis diminishes the significance of the comparison.

Most of the data included in the meta-analysis come from centers that are highly experienced in performing this type of surgery and different hybrid prostheses. Therefore, the results may vary according to center experience and may not be generalizable. The follow-up results were obtained from published Kaplan–Meier curves rather than obtained as true IPD, limiting the scope of analysis, particularly with regard to multivariable modeling and competing risks.

## 5. Conclusions

The comparison between AMDS and FET for the treatment of acute type A aortic dissection remains a subject of ongoing debate, primarily focused on their respective clinical outcomes and the strength of supporting evidence. FET is generally favored, owing to its association with lower mortality rates and more favorable long-term outcomes, supported by a robust body of clinical data. In contrast, AMDS represents a newer technique with limited evidence, and its safety and efficacy have yet to be definitively established.

## Figures and Tables

**Table 1:** Baseline patient’s characteristics *.

Variable	AMDS (N = 319)	FET (N = 4129)	*p*-Value
Mean age, years	60.8 ± 2.9	52.0 ± 5.2	**<0.001**
Male	70.5% (225/319)	77.6% (3205/4129)	**0.005**
Mean BMI	28.5 ± 1.2	26.0 ± 1.4	**0.023**
Connective tissue disease	0% (0/148)	7.1% (202/2832)	**0.001**
Diabetes	8.8% (24/273)	6.7% (244/3637)	0.234
COPD	7.1% (16/226)	5.6% (86/1544)	0.449
Hypertension	69.0% (220/319)	75.4% (2761/3663)	**0.014**
CKD	17.2% (55/319)	3.8% (79/2096)	**<0.001**
CAD	11.0% (30/273)	7.7% (123/1605)	0.082
Reintervention	3.7% (8/218)	3.5% (89/2528)	>0.999
Acute neurological deficit	29.4% (53/180)	5.9% (128/2186)	**<0.001**
Hemopericardium	12.3% (21/171)	17.1% (151/882)	0.146

BMI = body mass index; CAD = coronary artery disease; CKD = chronic kidney disease; COPD = chronic obstructive pulmonary disease; CPR = cardiopulmonary resuscitation. Bold means *p* < 0.05. * The dominator is based on the data availability among the included studies.

**Table 2:** Meta-analysis of the intraoperative data.

Outcome	Group	No. of Studies	No. of Patients	Estimate [95%CI]	Heterogeneity: I^2^, *p*-Value	Group Difference
Bilateral SACP	AMDS	5	273	54.0% [24.6–80.9]	92.2%, *p* < 0.0001	*p* = 0.587
	FET	20	2122	65.2% [38.5–84.9]	93.1%, *p* < 0.0001	
Hypothermic temperature	AMDS	4	180	27.2 °C [26.6–27.8]	91.4%, *p* < 0.0001	***p* < 0.001**
	FET	27	3454	23.9 °C [22.9–25.0]	99.8%, *p* < 0.0001	
Root replacement	AMDS	5	226	33.2% [22.0–46.7]	65.3%, *p* = 0.0213	*p* = 0.199
	FET	28	3474	26.8% [21.5–33.0]	89.6%, *p* < 0.0001	
VSRR	AMDS	4	180	31.8% [9.7–66.7]	84.1%, *p* = 0.0003	***p* = 0.001**
	FET	26	3321	3.2% [1.8–5.7]	85.5%, *p* < 0.0001	
Bentall	AMDS	4	180	5.4% [1.0–24.7]	62.1%, *p* = 0.0479	*p* = 0.171
	FET	26	3321	16.6% [12.5–21.6]	88.7%, *p* < 0.0001	
Root repair	AMDS	5	226	3.6% [0.3–35.8]	83.9%, *p* < 0.0001	***p* = 0.049**
	FET	27	3408	0.9% [0.3–2.5]	78.0%, *p* < 0.0001	
Wheat	AMDS	6	319	0.0% [0.0–0.0]	-	*p* = 0.920
	FET	28	3474	1.4% [0.9–2.3]	40.7%, *p* = 0.0142	
CABG	AMDS	6	319	7.2% [3.0–16.0]	66.0%, *p* = 0.0118	*p* = 0.930
	FET	28	3474	6.9% [4.3–10.8]	92.8%, *p* < 0.0001	
AV repair	AMDS	6	319	3.0% [0.4–18.3]	79.4%, *p* = 0.0002	*p* = 0.803
	FET	28	3474	3.9% [2.1–7.2]	91.2%, *p* < 0.0001	
AV replacement	AMDS	6	319	2.5% [0.3–18.1]	79.1%, *p* = 0.0002	*p* = 0.935
	FET	28	3474	2.7% [1.6–4.7]	81.4%, *p* < 0.0001	
MV surgery	AMDS	6	319	1.6% [0.5–4.3]	0.0%, *p* = 0.7141	*p* = 0.782
	FET	28	3474	1.3% [0.7–2.3]	56.7%, *p* = 0.0001	
CPB time	AMDS	4	180	238 min [189–299]	96.3%, *p* < 0.0001	*p* = 0.262
	FET	32	4129	204 min [192–217]	99.2%, *p* < 0.0001	
CXC time	AMDS	4	180	134 min [102–176]	96.0%; *p* < 0.0001	*p* = 0.394
	FET	30	3997	119 min [112–126]	98.5%, *p* < 0.0001	
CA time	AMDS	6	319	54 min [30–98]	99.6%, *p* < 0.0001	***p* = 0.047**
	FET	27	3645	29 min [24–35]	99.8%, *p* < 0.0001	
SACP time	AMDS	3	63	57 min [22–144]	98.3%, *p* < 0.0001	*p* = 0.869
	FET	14	1281	52 min [37–74]	99.9%, *p* < 0.0001	

AMDS = Ascyrus Medical Dissection Stent; AV = aortic valve; CA = circulatory arrest; CI = confidence interval; CPB = cardiopulmonary bypass; CXC = cross-clamp; FET = frozen elephant trunk; SACP = selective antegrade cerebral perfusion; VSRR = valve sparing root replacement. Bold means *p* < 0.05.

**Table 3:** Meta-analysis of the postoperative outcomes.

Outcome	Group	No. of Studies	No. of Patients	Estimate [95%CI]	Heterogeneity: I^2^, *p*-Value	Group Difference
Surgical bleeding	AMDS	4	180	21.2% [14.5–29.9]	17.7%, *p*= 0.3024	***p* < 0.001**
	FET	23	2867	6.4% [4.1–10.1]	87.7%, *p* < 0.0001	
Dialysis	AMDS	4	120	16.4% [10.6–24.3]	0.0%, *p* = 0.4049	*p* = 0.999
	FET	17	2096	16.4% [11.8–22.3]	88.0%, *p* < 0.0001	
CVA	AMDS	6	319	8.8% [4.9–15.4]	47.3%, *p* = 0.0909	*p* = 0.717
	FET	19	2100	7.7% [5.1–11.7]	80.5%, *p* < 0.0001	
SCI	AMDS	3	111	0.0% [0.0–0.0]	-	*p* = 0.178
	FET	19	2207	5.2% [3.6–7.3]	56.6%, *p* = 0.0013	
ICU time	AMDS	6	319	7.8 days [6.1–10.0]	74.4%, *p* = 0.0015	*p* = 0.599
	FET	23	3446	7.0 days [5.2–9.5]	99.5%, *p* < 0.0001	
LOS time	AMDS	5	213	14.8 days [13.3–16.4]	14.3%, *p* = 0.3232	***p* < 0.001**
	FET	20	2420	19.2 days [17.3–21.4]	98.0%, *p* < 0.0001	
Hospital mortality	AMDS	6	319	14.5% [11.0–18.9]	0.0%, *p* = 0.9751	***p* = 0.037**
	FET	31	3917	10.0% [8.1–12.3]	73.9%, *p* = 0.0012	
Follow-up time	AMDS	3	196	0.8 yr [0.2–3.6]	99.7%, *p* < 0.0001	*p* = 0.218
	FET	18	1520	2.1 yr [1.6–2.8]	99.8%, *p* < 0.0001	
Late death	AMDS	3	196	7.8%/yr [1.6–36.8]	84.7%, *p* = 0.0014	*p* = 0.285
	FET	17	1472	3.3%/yr [2.4–4.4]	43.0%, *p* = 0.0311	

AMDS = Ascyrus Medical Dissection Stent; CI = confidence interval; CVA = cerebrovascular accident; FET = frozen elephant trunk; ICU = intensive care unit; LOS = length of stay; PND = permanent neurological dysfunction; SCI = spinal cord injury; TND = temporary neurological dysfunction; yr = years. Bold means *p* < 0.05.

**Table 4:** Univariable meta-regression on hospital mortality.

	AMDS	FET
Variable	OR (95%CI), *p*-Value	OR (95%CI), *p*-Value
Mean age, years	1.06 (0.92–1.22), *p* = 0.408	1.01 (0.98–1.05), *p* = 0.484
Male %	0.98 (0.93–1.02), *p* = 0.314	1.01 (0.98–1.03), *p* = 0.586
Mean BMI	0.75 (0.53–1.05), *p* = 0.095	1.05 (0.76–1.44), *p* = 0.779
Connective tissue disease %	No patient	0.98 (0.95–1.02), *p* = 0.284
Diabetes %	0.92 (0.83–1.03), *p* = 0.144	0.99 (0.96–1.02), *p* = 0.565
COPD %	0.95 (0.85–1.07), *p* = 0.424	1.01 (0.93–1.09), *p* = 0.824
Hypertension %	1.02 (0.99–1.04), *p* = 0.142	0.98 (0.96–1.01), *p* = 0.309
CKD %	0.97 (0.93–1.01), *p* = 0.150	1.04 (0.93–1.16), *p* = 0.522
CAD %	0.96 (0.83–1.11), *p* = 0.606	0.95 (0.90–1.01), *p* = 0.129
Reintervention %	0.72 (0.19–2.69), *p* = 0.629	1.05 (0.92–1.20), *p* = 0.434
Acute neurological deficit %	1.03 (0.94–1.13), *p* = 0.582	0.97 (0.91–1.04), *p* = 0.449
Hemopericardium %	0.98 (0.92–1.05), *p* = 0.647	1.01 (0.98–1.04), *p* = 0.571
BSACP %	0.99 (0.98–1.01), *p* = 0.321	1.00 (0.99–1.01), *p* = 0.731

BMI = body mass index; BSACP = bilateral selective antegrade cerebral perfusion; CAD = coronary artery disease; CI = confidence interval; CKD = chronic kidney disease; COPD = chronic obstructive pulmonary disease; CPR = cardiopulmonary resuscitation; OR = odds ratio.

## Data Availability

The data that support the findings of this study are available from the corresponding Author upon reasonable request.

## Supplementary Materials

The following supporting information can be downloaded at: https://www.mdpi.com/article/10.3390/jcm14145170/s1, Figure S1: Schoenfeld residual test on overall survival; Table S1: Search strategy; Table S2: Outline of the included studies; Table S3: Risk of Bias in Non-Randomized Studies of Interventions (ROBINS-I) with traffic lights.

## Abbreviations

The following abbreviations are used in this manuscript:

AMDS	Ascyrus Medical Dissection Stent
ATAAD	Acute Type A Aortic Dissection
CI	Confidence Interval
DANE	Distal Anastomotic New Entry
FET	Frozen Elephant Trunk
HR	Hazard Ratio
IPD	Individual Patient Data
IR	Incidence Rate
OR	Odds Ratio
PEM	Pooled Estimated Mean
PER	Pooled Estimated Rate
TAR	Total Arch Replacement
