# Supplementary material for: Total Arch Replacement with Ascyrus Medical Dissection Stent Versus Frozen Elephant Trunk in Acute Type A Aortic Dissection: A Meta-Analysis
Source: J Clin Med. 2025 Jul 21;14(14):5170. doi: 10.3390/jcm14145170 (PMC12296124; doi:10.3390/jcm14145170)

Table S1 – Search strategy

#	Search
1	("freezing"[MeSH Terms] OR "freezing"[All Fields] OR "frozen"[All Fields]) AND ("elephant s"[All Fields] OR "elephants"[MeSH Terms] OR "elephants"[All Fields] OR "elephant"[All Fields]) AND ("torso"[MeSH Terms] OR "torso"[All Fields] OR "trunk"[All Fields] OR "trunk s"[All Fields] OR "trunks"[All Fields])
2	"ascyrus"[All Fields] AND ("medic"[All Fields] OR "medical"[All Fields] OR "medicalization"[MeSH Terms] OR "medicalization"[All Fields] OR "medicalizations"[All Fields] OR "medicalize"[All Fields] OR "medicalized"[All Fields] OR "medicalizes"[All Fields] OR "medicalizing"[All Fields] OR "medically"[All Fields] OR "medicals"[All Fields] OR "medicated"[All Fields] OR "medication s"[All Fields] OR "medics"[All Fields] OR "pharmaceutical preparations"[MeSH Terms] OR ("pharmaceutical"[All Fields] AND "preparations"[All Fields]) OR "pharmaceutical preparations"[All Fields] OR "medication"[All Fields] OR "medications"[All Fields]) AND ("dissect"[All Fields] OR "dissected"[All Fields] OR "dissecting"[All Fields] OR "dissection"[MeSH Terms] OR "dissection"[All Fields] OR "dissections"[All Fields] OR "dissects"[All Fields]) AND ("stent s"[All Fields] OR "stentings"[All Fields] OR "stents"[MeSH Terms] OR "stents"[All Fields] OR "stent"[All Fields] OR "stented"[All Fields] OR "stenting"[All Fields])
3	1 OR 2
4	"aorta, thoracic"[MeSH Terms] OR ("aorta"[All Fields] AND "thoracic"[All Fields]) OR "thoracic aorta"[All Fields] OR ("aortic"[All Fields] AND "arch"[All Fields]) OR "aortic arch"[All Fields]
5	"aortic dissection"[MeSH Terms] OR ("aortic"[All Fields] AND "dissection"[All Fields]) OR "aortic dissection"[All Fields]
7	4 OR 5
8	3 AND 7

Table S2 – Outline of the included studies

Paper	Institution	Country	Study period	Stud y	Prosthesi s
Bozso 2022	University of Alberta, Edmonton, Alberta; Western University, London, Ontario; Montreal Heart Institute, Montreal, Quebec; University of Toronto, Toronto, Ontario, Canada; German Heart Centre, Berlin	Canada, Germany	Mar 2017 - Jan 2019	PRO	AMDS
Luehr 2023	Heart Centre, University of Cologne, Cologne; Goethe University and University Hospital Frankfurt, Frankfurt	Germany	Jan 2020 - Jun 2022	OBS	AMDS
Mehdiani 2022	Heinrich Heine University Duesseldorf, Düsseldorf	Germany	Aug 2019 - Dec 2020	OBS	AMDS
Immohr 2023	University Hospital Düsseldorf, Heinrich-Heine-University Düsseldorf, Düsseldorf	Germany	Aug 2019 - Dec 2020	OBS	AMDS
Pitts 2025	German Heart Center, Berlin	Germany	2018-2022	PSM	AMDS
Szeto 2024	26 Centers	International	Jul 2022 - Nov 2023	PRO	AMDS
Azuma 2023	Kyoto Katsura Hospital, Kyoto	Japan	Sep 2014 - Mar 2022	OBS	FET
Chabry 2021	21 Centers	France	Apr 2016 - Apr 2019	OBS	FET
Chen 2010	Nanjing Cardiovascular Disease Research Institute, Nanjing First Hospital Affiliated to Nanjing Medical University, Nanjing	China	Aug 2004 - May 2009	OBS	FET
Chivasso 2022	University Hospital San Giovanni di Dio e Ruggi d'Aragona, Salerno	Italy	Dec 2017 - Jan 2022	OBS	FET
Cuko 2023	Hopital Cardiologique de Haut-Leveque, Bordeaux University Hospital	France	Aug 2018 - Aug 2022	OBS	FET
Dai 2022	Fuwai Hospital, National Center for Cardiovascular Diseases, Chinese Academy of Medical Sciences and Peking Union Medical College, Beijing	China	Aug 2020 - Sep 2020	OBS	FET
Dohle 2023	Johannes-Gutenberg University, Mainz	Germany	Jun 2017 - Apr 2021	OBS	FET
Hoffman 2013	University Hospital of RWTH Aachen, Aachen	Germany	Nov 2009 - Sep 2011	OBS	FET
Hu 2017	Renmin Hospital of Wuhan University, Wuhan, Hubei	China	Aug 2008 - Dec 2015	OBS	FET
Huang F 2022	Fujian Provincial Hospital, Fuzhou	China	Jan 2017 - Dec 2019	OBS	FET
Jiang 2020	General Hospital of Northern Theater Command, Shenyang, Liaoning	China	May 2016 - Apr 2018	OBS	FET

Kong 2022	The First Affiliated Hospital of USTC, University of Science and Technology of China (USTC), Hefei, Anhui	China	Oct 2020 - Nov 2021	OBS	FET
Liu 2022	General Hospital of Northern Theater Command, Shenyang, Liaoning	China	Oct 2019 - Jul 2021	OBS	FET
Ma 2018	Tongji Hospital, Tongji Medical College, Huazhong University of Science and Technology, Wuhan	China	Jan 2013 - Dec 2015	OBS	FET
Mariscalco 2019	Glenfield Hospital, University Hospitals of Leicester NHS Trust, Leicester; Manchester Royal Infirmary, Manchester; Papworth Hospital, Papworth; Liverpool Heart and Chest Hospital, Liverpool; University Hospital Birmingham NHS Foundation Trust, Birmingham; Barts Heart Center, St Bartholomew's Hospital, London; Royal Brompton Hospital, London; Derriford Hospital, Plymouth; University Hospital Southampton NHS Foundation Trust, Southampton	UK	Jun 2013 - Oct 2017	OBS	FET
Shen 2022	The Second Xiangya Hospital of Central South University, Changsha	China	May 2017 - Dec 2020	OBS	FET
Shi 2014	First Affiliated Hospital, China Medical University, Shenyang	China	Jan 2006 - Dec 2011	OBS	FET
Shi 2020	Renmin Hospital of Wuhan University, Wuhan, Hubei	China	Jan 2017 - Jan 2019	PSM	FET
Shrestha 2015	Hannover Medical School, Hannover	Germany	Aug 2001 - Mar 2013	OBS	FET
Tong 2022	Guangdong Cardiovascular Institute, Guangdong Provincial People's Hospital, Guangdong Academy of Medical Sciences, Guangzhou	China	Jan 2013 - Nov 2020	PSM	FET
Wisniewski 2022	University Hospital Muenster, Muenster	Germany	Apr 2015 - Jul 2021	OBS	FET
Wu B 2014	Shanghai Institute of Cardiovascular Diseases, Zhongshan Hospital, Fudan University, Shanghai	China	Jan 2008 - Dec 2012	OBS	FET
Xiao 2014	West China Hospital, Sichuan University Chengdu, Sichuan	China	Feb 2008 - Dec 2011	OBS	FET
Yang C 2022	Xijing Hospital, Fourth Military Medical University, Xi'an	China	Dec 2017 - Jan 2020	OBS	FET
Zhang 2014	Changhai Hospital, Second Military Medical University, Shanghai	China	Jan 2002 - Jun 2010	OBS	FET
Dong 2023	The First Affiliated Hospital of Nanjing Medical University, Nanjing	China	Jan 2020 - Dec 2022	OBS	FET

Gao 2024	First Affiliated Hospital of Gannan Medical University	China	Jan 2018 - Jun 2021	OBS	FET
King 2023	University of Colorado, Denver, Colorado	USA	2015 - 2021	OBS	FET
Takagi 2024	Toyohashi Heart Center, Gobudori, Oyama-Cho, Toyohashi	Japan	2007 - 2021	OBS	FET
Wei 2024	Fuwai Hospital, National Center for Cardiovascular Disease, Chinese Academy of Medical Sciences and Peking Union Medical College, Beijing	China	Jan 2013 - Dec 2018	OBS	FET
Xiang 2024	Affiliated Hospital of North Sichuan Medical College	China	Jan 2018 - Oct 2020	PSM	FET
Yan 2024	Fuwai Hospital, National Center for Cardiovascular Disease, Chinese Academy of Medical Sciences and Peking Union Medical College, Beijing	China	Jan 2021 - Aug 2022	OBS	FET

AMDS = Ascyrus Medical Dissection Stent; FET = frozen elephant trunk; OBS = observational; PRO = prospective; PSM = propensity score matched

Table S3 – Risk of Bias in Non-Randomized Studies of Interventions (ROBINS-I) with traffic lights

Study	D1	D2	D3	D4	D5	D6	D7	Overall
Bozso 2022	⊖	⊖	⊕	⊕	⊖	⊕	⊖	⊖
Luehr 2023	⊖	⊖	⊕	⊕	⊖	⊕	⊖	⊖
Mehdiani 2022	⊗	⊖	⊕	⊕	⊖	⊕	⊖	⊗
Immohr 2023	⊖	⊖	⊕	⊕	⊖	⊕	⊖	⊖
Pitts 2025	⊕	⊕	⊕	⊕	⊕	⊕	⊕	⊕
Szeto 2024	⊖	⊖	⊕	⊕	⊖	⊕	⊖	⊖
Azuma 2023	⊗	⊖	⊕	⊕	⊖	⊕	⊖	⊗
Chabry 2021	⊖	⊖	⊕	⊕	⊖	⊕	⊖	⊖
Chen 2010	⊖	⊖	⊕	⊕	⊖	⊕	⊖	⊖
Chivasso 2022	⊖	⊕	⊕	⊕	⊖	⊕	⊖	⊖
Cuko 2023	⊖	⊖	⊕	⊕	⊖	⊕	⊖	⊖
Dai 2022	⊗	⊖	⊕	⊕	⊖	⊕	⊖	⊗
Dohle 2023	⊖	⊖	⊕	⊕	⊖	⊕	⊖	⊖
Hoffman 2013	⊖	⊖	⊕	⊕	⊖	⊕	⊖	⊖
Hu 2017	⊗	⊖	⊕	⊕	⊖	⊕	⊖	⊗
Huang F 2022	⊖	⊖	⊕	⊕	⊖	⊕	⊖	⊖
Jiang 2020	⊖	⊖	⊕	⊕	⊖	⊕	⊖	⊖
Kong 2022	⊗	⊖	⊕	⊕	⊖	⊕	⊖	⊗
Liu 2022	⊖	⊖	⊕	⊕	⊖	⊕	⊖	⊖
Ma 2018	⊗	⊖	⊕	⊕	⊖	⊕	⊖	⊗
Mariscalco 2019	⊖	⊖	⊕	⊕	⊖	⊕	⊖	⊖
Shen 2022	⊖	⊖	⊕	⊕	⊖	⊕	⊖	⊖
Shi 2014	⊖	⊖	⊕	⊕	⊖	⊕	⊖	⊖
Shi 2020	⊕	⊕	⊕	⊕	⊕	⊕	⊕	⊕
Shrestha 2015	⊖	⊖	⊕	⊕	⊖	⊕	⊖	⊖
Tong 2022	⊕	⊕	⊕	⊕	⊕	⊕	⊕	⊕
Wisniewski 2022	⊖	⊖	⊕	⊕	⊖	⊕	⊖	⊖
Wu B 2014	⊖	⊖	⊕	⊕	⊖	⊕	⊖	⊖
Xiao 2014	⊖	⊖	⊕	⊕	⊖	⊕	⊖	⊖
Yang C 2022	⊖	⊖	⊕	⊕	⊖	⊕	⊖	⊖
Zhang 2014	⊗	⊖	⊕	⊕	⊖	⊕	⊖	⊗
Dong 2023	⊕	⊖	⊕	⊕	⊖	⊕	⊖	⊖
Gao 2024	⊗	⊖	⊕	⊕	⊖	⊕	⊖	⊗

King 2023	⊗	⊖	⊕	⊕	⊖	⊕	⊖	⊗
Takagi 2024	⊗	⊖	⊕	⊕	⊖	⊕	⊖	⊗
Wei 2024	⊖	⊖	⊕	⊕	⊖	⊕	⊖	⊖
Xiang 2024	⊕	⊕	⊕	⊕	⊕	⊕	⊕	⊕
Yan 2024	⊖	⊖	⊕	⊕	⊖	⊕	⊖	⊖

D1 = bias due to confounding; D2 = bias due to selection of participants; D3 = bias in classification of interventions; D4 = bias due to deviation from intended interventions; D5 = bias due to missing data; D6 = bias in measurements of outcomes; D7 = bias in selection of the reported results. ⊕ = low bias; ⊖ = moderate bias; ⊗ = serious bias.

Figure S1 – Schoenfeld residual test on overall survival

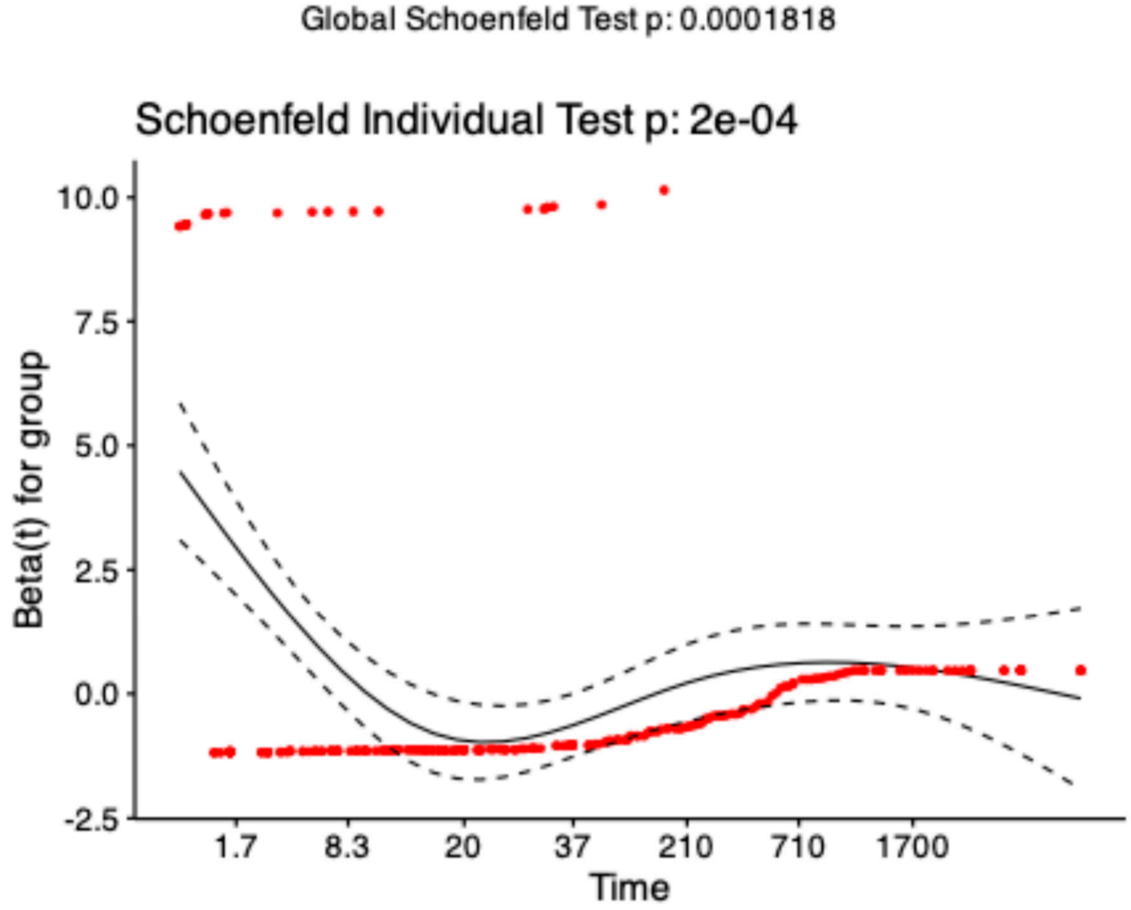

Supplement: Supplementary file 1 [file jcm-14-05170-s001.zip › jcm-3748465-supplementary.pdf]
